# Distinguishing Profiles of Extraction Versus Non-extraction Cases Post-orthodontic Treatment: A Cross-Sectional Study

**DOI:** 10.7759/cureus.71787

**Published:** 2024-10-18

**Authors:** Mohammad Qali, Samaa Alsaraf, Hashem Alsaegh

**Affiliations:** 1 Surgical Sciences, College of Dentistry, Health Sciences Center, Kuwait University, Kuwait, KWT; 2 Dentistry, Ministry of Health, Kuwait, KWT

**Keywords:** dental, dentists, extraction, orthodontics, profile

## Abstract

Objectives: It has been proposed that ideal esthetics are executed once the face, arch, and tooth forms are in consensus. While the profile can provide valuable insights into orthodontic treatment outcomes, it may not always be sufficient to definitively differentiate between cases treated with and without extractions. This raises the aim of the study to assess if dentists are able to differentiate between extraction and non-extraction orthodontic cases depending on the profile photographs of patients post-orthodontic treatment.

Materials and methods: This study was a retrospective cross-sectional survey among general dentists and dental specialists. A sample of 151 participants was approached through a multi-stage random sampling method. The statistical analysis for the study was conducted using chi-square tests of goodness of fit, Pearson chi-square, and the McNemar test.

Results: Both orthodontists and other specialties performed significantly well in characterizing the cases. Orthodontists also performed significantly better than other dental specialties.

Conclusion: The present study challenges the initial hypothesis that dentists are unable to identify tooth extraction based on photographs. The findings reveal that both orthodontists and dentists from other specialties demonstrated a statistically significant ability to identify these characteristics from photographic evidence.

## Introduction

Dentofacial aesthetics is an essential aspect of the orthodontic treatment we provide. It has been proposed that ideal esthetics are executed once the face, arch, and tooth forms are in consensus [1]. This is corroborated by the macroesthetic, miniesthetic, and microesthetic categorizations of the Aesthetic Dentofacial Analysis by Dr. David Sarver. Macroesthetics takes into consideration the facial harmony and balance [2]. Miniesthetics interprets the tooth-lip relationship along with the incisal and gingival display, buccal corridor, and smile arc of the maxillary anterior teeth following the lower lip. Microesthetics portray the tooth morphology, shade, and color [3]. Nowadays, society and media are displaying an esthetic pattern that people are now following [4].

The study’s main focus is macroesthetics. While the profile can provide valuable insights into orthodontic treatment outcomes, it may not always be sufficient to definitively differentiate between cases treated with and without extractions.

In some cases, the absence of extractions may result in a fuller appearance in the lips and cheeks due to the presence of all teeth. Conversely, extraction cases may result in a flatter profile due to the retraction of anterior teeth. However, individual variations in facial anatomy, muscle tone, and soft tissue characteristics can influence the final profile appearance.

This debate began years ago when Dr. Edward Angle, the father of orthodontics, relied on expansion to gain space in the dental arch, which led to muscular imbalances and full profiles. Later on, Dr. Charles Tweed, a student of Angle, was frustrated upon recalling Angle’s patients as their profiles were full and protrusive with an unstable dentition. Tweed popularized extractions for all cases, whereas many orthodontists feared the effect of extractions on the soft tissue profile and the appearance of a dished-in face. After that, Dr. Sarver came up with the aforementioned Aesthetic Dentofacial Analysis to guide orthodontists to study the full picture by keeping into consideration the soft tissues and overall facial harmony and balance before deciding whether to extract or not and develop a proper treatment plan specific to that patient, minimizing unwanted results. This raises the aim of the study to assess if dentists are able to differentiate between extraction and non-extraction orthodontic cases depending on the profile photographs of patients post-orthodontic treatment.

The hypothesis of our study is that dentists cannot differentiate by looking at extraoral frontal and lateral profile views of patients who had extractions during their orthodontic treatment, given that their orthodontic treatment was carefully planned, executed, and tailored to the patient.

## Materials and methods

Study design and selection of participants

This study was a retrospective cross-sectional survey among general dentists and dental specialists. A sample of 151 participants who are working at Kuwait University and 10 government sites including specialty centers and polyclinics of the Ministry of Health, Kuwait, were approached through a multi-stage random sampling method. Data were collected through personal visits to each of the public dental centers for a period of two months January and February 2024. The inclusion criteria for this study consist of dentists currently practicing at Kuwait University and governmental specialized dental centers in Kuwait. The exclusion criteria include dental students and non-dental professionals (laypersons).

Of these selected 151, a sample of 10 participants were randomly selected to repeat their participation and answer the questionnaire a second time two months after their initial partaking.

The objectives of this were to compare the dentists' current performance to chance for both tasks, to compare their previous performance to chance, and to compare their previous and current performances using the McNemar test to determine if any significant differences existed.

Sample size considerations

In 2021, there were approximately 1871 dentists working in the government's public health sector, according to the latest manpower statistics (Ministry of Health, 2021). We estimated that sampling about 10% of this population would allow us to generalize our findings to the entire population of dentists working for the Ministry of Health. The random selection of polyclinics was performed using a computer-based random number generator. We also included faculty professors from Kuwait University - College of Dentistry, Kuwait.

Data collection

The period of data collection lasted four months, from January to April 2024. We carried out the survey, targeting dentists. Earlier permission from the chief dentist at each clinic/center was obtained and the above-mentioned dentists checked the completion of each questionnaire. The average time required to complete the questionnaire was five minutes, but due to the high number of patients in some clinics, some participants required a longer duration of time.

Ethical considerations

Ethical consent was obtained from the Ministry of Health as well as the Kuwait University - College of Dentistry's Research Ethical Review Committee. The ethics form provides information regarding the type of the research, the population being studied, and any special considerations (e.g., if any invasive procedures will be implemented in the research). Approval for access to Ministry of Health facilities was also granted by the Ministry of Health ethics committee. The only item of ethical relevance, information that may personally identify the dentist, was excluded from the questionnaire.

Survey instrument

A two-page questionnaire of 20 questions was designed to allow dentists to evaluate presented cases. Cases used in this study were treated by the principle investigator and the order in which the cases were presented was randomized. Participants/dentists were asked about their wish to participate in this study and mention their specialty (General Dental Practitioner, Orthodontist, Periodontist, Prosthodontist, Endodontist, Advanced General Dentist, Oral Maxillofacial Surgeon, Pedodontist, Oral Maxillofacial Radiologist, Residents of the Kuwaiti Board of Orthodontics and Dentofacial Orthopedics). The 20 cases were extraoral frontal photographs (at rest and smiling) and lateral profile views of patients who completed their orthodontic treatment and participants were asked to differentiate which cases were treated with extractions during their orthodontic treatment.

Statistical analysis

The statistical analysis for the study was conducted using two primary methods: chi-square tests of goodness of fit and Pearson chi-square. The chi-square goodness of fit tests were applied to assess the within-group ability of dentists from various specialties to identify orthodontic extraction cases from photographs taken post-treatment. Pearson's chi-square test was used for between-group comparisons to determine if there were significant differences in identification abilities across the different dental specialties.

The McNemar test was used to compare the dentists' performance between the first and second-time points for the 10 dentists that repeated their contribution.

## Results

The results were analyzed separately for orthodontists and dentists from all other specialties combined as shown in Table 1. For the orthodontists' group consisting of 52 orthodontists (n = 628) 60.4% of the responses correctly identified cases of tooth extraction from photographs, while (n = 412) 39.6% were incorrect. The within-group p-value was less than 0.001, indicating that the orthodontists' ability to identify tooth extractions was statistically significant (Table 1).

**Table 1:** Dentists' ability to identify whether the patients did extraction or not based on photographs p-value > 0.05 is considered non-significant

	Were dentists able to identify whether the patient had extraction or not based on photographs shown				Within group p-value	Among group p-value
	No		Yes			
	N	N%	N	N%		
Orthodontists	412	39.6%	628	60.4%	<0.001	0.006
All other specialties	888	44.8%	1092	55.2%	<0.001	

**Figure 1:**
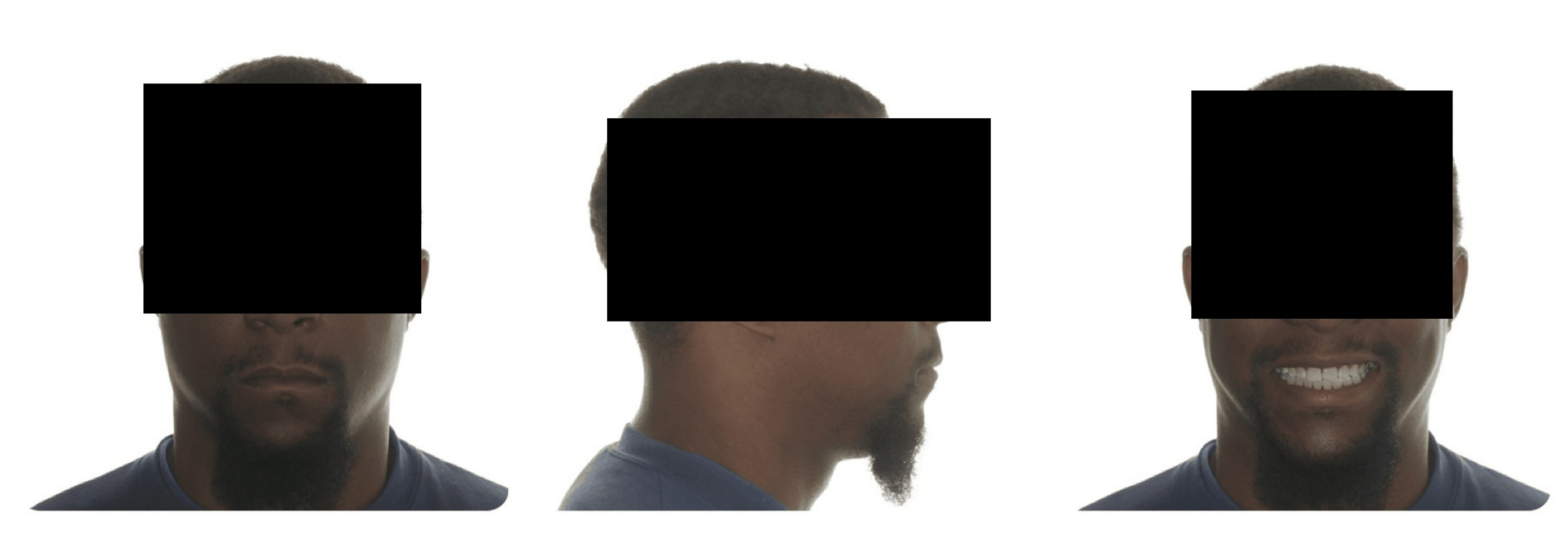
Frontal and lateral view post-orthodontic treatment with extractions - case that was labeled "incorrectly" (case no. 9 - Table 2)

**Figure 2:**
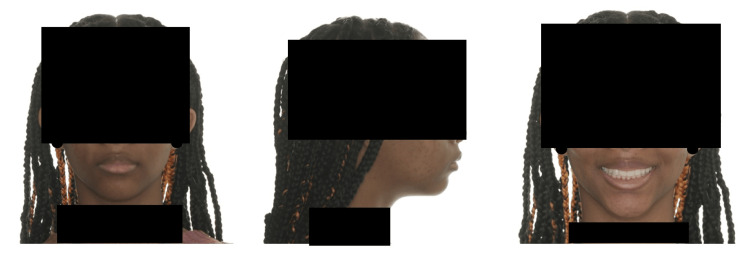
Frontal and lateral view post-orthodontic treatment without extractions - case that was labeled "correctly" (case no. 2 - Table 2)

In the group comprising all other dental specialties consisting of 99 dentists (n = 1092) 55.2% of the responses were correct, and (n = 888) 44.8% were incorrect. Similar to the orthodontists' group, the within-group p-value was less than 0.001, suggesting that dentists from other subspecialties could also identify tooth extractions from photographs with statistical significance. However, when comparing the two groups, the among-group p-value of 0.006 revealed a statistically significant difference in their performance. This implies that orthodontists were better at identifying tooth extractions from photographs compared to dentists from other specialties. Table 2 shows the number of dentists who were able to identify each case correctly. Figure 1 shows the cases that were most of the time labeled incorrectly by dentists and Figure 2 presents the most cases labeled correctly (Tables 1-2, Figures 1-2).

**Table 2:** The number (percentage) of dentists that were able to identify each case correctly

Case no.	Were all dentists able to identify extraction based on the picture shown?	
	No	Yes
	N (N%)	N (N%)
1	67 (44.3%)	84 (55.6%)
2	40 (26.4%)	111 (73.5%)
3	48 (31.7%)	103 (68.2%)
4	65 (43.0%)	86 (56.9%)
5	98 (64.9%)	53 (35.0%)
6	48 (31.7%)	103 (68.2%)
7	72 (47.6%)	79 (52.3%)
8	69 (45.6%)	82 (54.3%)
9	99 (65.5%)	52 (34.4%)
10	73 (48.3%)	78 (51.6%)
11	49 (32.4%)	102 (67.5%)
12	92 (60.9%)	59 (39.0%)
13	85 (56.2%)	66 (43.7%)
14	73 (48.3%)	78 (51.6%)
15	73 (48.3%)	78 (51.6%)
16	48 (31.7%)	103 (68.2%)
17	69 (45.6%)	82 (54.3%)
18	42 (27.8%)	109 (72.1%)
19	41 (27.1%)	110 (72.8%)
20	49 (32.4%)	102 (67.5%)

Table 3 presents the results of 10 dentists who were asked for the second time to identify whether patients had undergone tooth extractions or not, based on photographs. This task was performed at two different time points.

**Table 3:** Dentists’ performance in identifying tooth extractions from photographs

		N	N%
Were the dentists able to identify whether the patient had extraction or not the 2nd time	No	89	44.5%
	Yes	111	55.5%
Were the dentists able to identify whether the patient had extraction or not the 1st time	No	99	49.5%
	Yes	101	50.5%

During the first time point, out of the 200 answers/inputs (10 dentists x 20 cases), 101 inputs (50.5%) correctly identified if the case had undergone an extraction or not. The remaining 99 entries (49.5%) were incorrectly identified. In the second time point, the dentists' performance improved slightly. Out of the total inputs, 111 (55.5%) were correctly identified as having undergone an extraction, while 89 (44.5%) were incorrectly identified (Table 3).

Table 4 shows the responses at the first time point, the observed proportion of correct identifications (50.5%) was compared to a test proportion of 0.5 (50%), which represents the expected proportion under the null hypothesis of random guessing. The results of the statistical test yielded a p-value of 0.944. This p-value suggests that the observed proportion of correct identifications by the dentists was not significantly different from the chance level of 50%. In other words, the dentists' ability to correctly identify tooth extractions from photographs during the first time point was not significantly better than random guessing (Table 4).

**Table 4:** Dentists' performance in identifying tooth extractions - first attempt p-value > 0.05 is considered non-significant. N/A: not applicable

	Category	N	Observed prop.	Test prop.	p-value
Were the dentists able to identify whether the patient had extraction or not the 1st time	Yes	101	0.51	0.50	0.944
	No	99	0.50	N/A	N/A

Table 5 shows the responses during the second time point of the study, the dentists were again asked to identify whether patients had undergone tooth extractions or not, based on photographs. The observed proportion of correct identifications (55.5%) was compared to a test proportion of 0.5 (50%), which represents the expected proportion under the null hypothesis of random guessing. The results of the statistical test yielded a p-value of 0.137. This p-value suggests that the observed proportion of correct identifications by the dentists was not statistically significantly different from the chance level of 50% (Table 5).

**Table 5:** Dentists' performance in identifying tooth extractions - second attempt p-value > 0.05 is considered non-significant. N/A: not applicable

	Category	N	Observed prop.	Test prop.	p-value
Were the dentists able to identify whether the patient had extraction or not the 2nd time	Yes	111	0.56	0.50	0.137
	No	89	0.44	N/A	N/A

The McNemar test was used to compare the dentists' performance between the first- and second-time points. Table 6 shows the cross-tabulation of the dentists' responses at the two-time points.

**Table 6:** McNemar test results: dentists' performance in identifying tooth extractions across two time points p-value > 0.05 is considered non-significant.

		Were the dentists able to identify whether patient had extraction or not the 2nd time		p-value (McNemar test)
		No	Yes	
		N (N%)	N (N%)	
Were the dentists able to identify whether patient had extraction or not the 1st time	No	40 (44.9%)	59 (53.2%)	0.387
	Yes	49 (55.1%)	52 (46.8%)	

Out of the 89 images that were incorrectly identified in the second time point, 40 images (44.9%) were also incorrectly identified in the first time point, while 49 images (55.1%) were correctly identified in the first time point. Out of the 111 images that were correctly identified in the second time point, 59 images (53.2%) were incorrectly identified in the first time point, while 52 images (46.8%) were correctly identified in the first time point.

**Figure 3:**
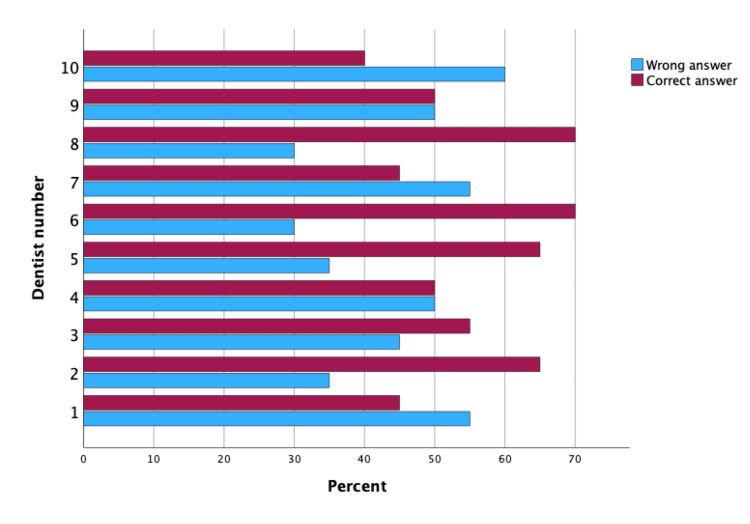
Clustered bar chart for frequency of correct and incorrect answers for each dentist in identifying tooth extractions cases at second time point

**Figure 4:**
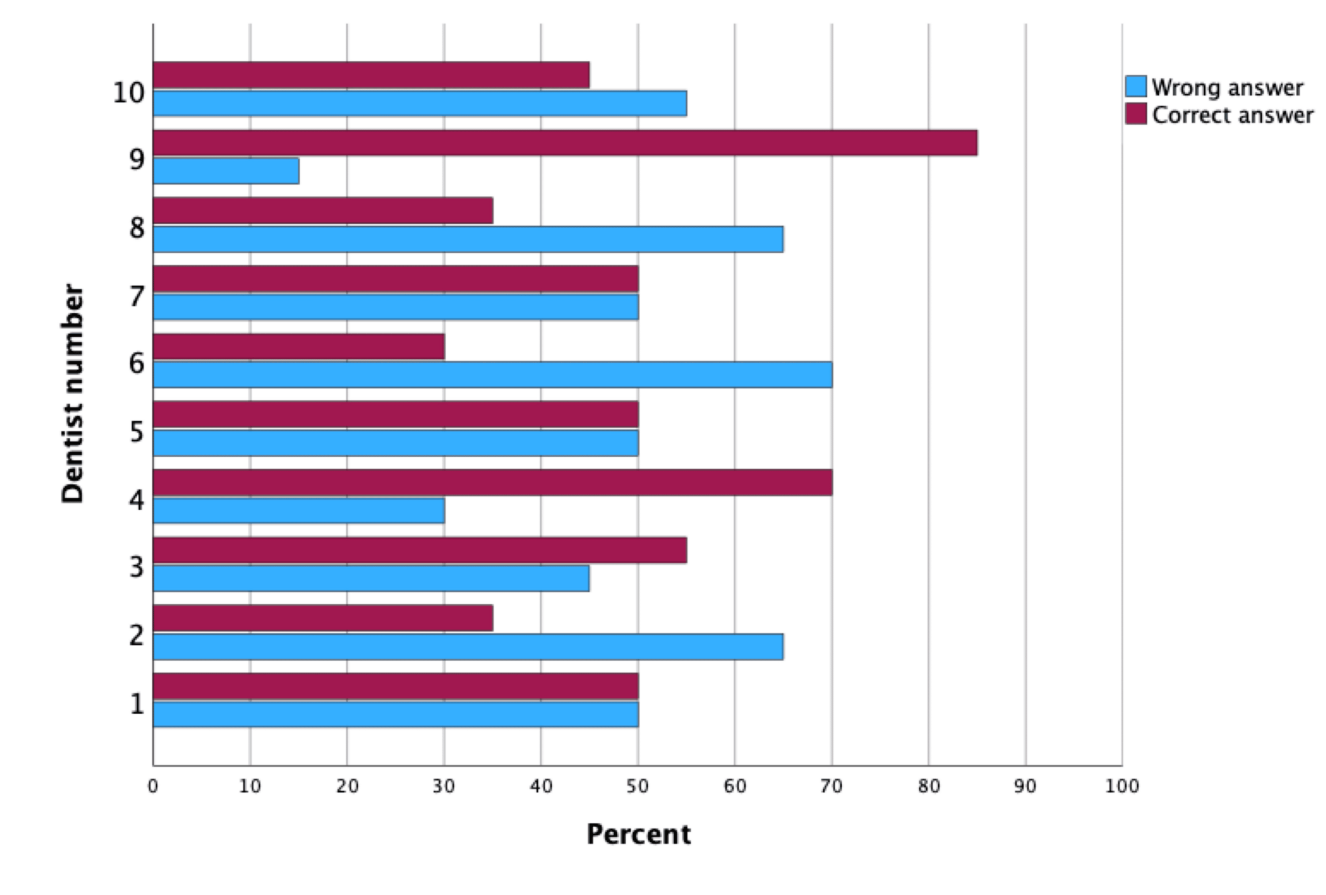
Clustered bar chart for frequency of correct and incorrect answers for each dentist in identifying tooth extractions cases at first time point

The p-value of the McNemar test was 0.387, which suggests that the difference in the dentists' performance between the two-time points was not statistically significant. In other words, while the dentists' overall performance improved slightly in the second time point, the difference was not statistically significant when compared to their performance in the first time point. Figures 3-4 are clustered bar charts highlighting the difference in the percentage of correct and incorrect answers by each of the 10 dentists participating twice in this study (Table 6, Figures 3-4).

## Discussion

The decision to extract premolars in orthodontics is to resolve tooth size-arch discrepancy, which may be followed by soft tissue profile changes. This may be a concern if beauty and harmony are affected. A raised question was to see if dentists can differentiate between cases treated with extraction and those without. In previous studies, Stephens et al. in 2005 and Erdinc et al. in 2007 supported the fact that orthodontists and general dentists were unable to differentiate between the facial profiles of patients that had extractions and those that did not [5,6]. However, in our study, both orthodontists and other specialties performed significantly well in characterizing the cases. Orthodontists also performed significantly better than other dental specialties. A possible reason behind this may be that orthodontists have higher expertise in macroesthetics of the face. Also, attaining general harmony and balance among various facial features is the orthodontist’s responsibility when predicting the patient's response to their treatment. Random selection of cases that were used in the study can introduce variability in the degree of soft tissue changes observed post-treatment. Factors such as initial lip thickness, muscle tone, and overall facial morphology can influence how soft tissues respond to orthodontic treatment, including extractions.

In cases where there are significant changes in the profile, such as those with thicker lips or different facial proportions, the amount of soft tissue retraction may vary compared to cases with thinner lips or different facial profiles. Predicting the exact amount of soft tissue retraction solely based on the profile can be challenging due to these individual variations. A study by Williams and Hosila showed that orthodontic treatment with extraction of premolars was followed by changes in their soft tissue profile; these changes were aesthetic and improved the profile, while in other cases they resulted in an unappealing outcome [7]. This demonstrates the importance of drawing out a good diagnosis scheme and a treatment plan to extract specific teeth and managing the extraction space properly to have a balanced facial esthetic instead of devastating it [8].

Although dentists could significantly identify extraction and non-extraction cases, a large number of the cases were labeled incorrectly. This shows that even though dentists can identify extraction from non-extraction cases, this is not true every time. This was also demonstrated in a study by Boley et al., which showed that dental professionals could determine only 50% of the time if a patient was treated with extraction [9]. Table 2 demonstrates the number (and percentage) of dentists that tagged each case correctly as extraction or non-extraction cases. Figure 1 shows most cases that were incorrectly labeled as a non-extraction case. This may be due to the racial background of the patient and that even with extractions, a full lip profile still exists. Figure 2 reveals the highest case that was marked correctly as a non-extraction case. It may follow the same reasoning as previously mentioned that full lips and the acute nasolabial angle may be the logic behind classifying both cases as non-extraction cases.

A larger sample size would help with more accurate and precise results, with more samples from other specialties to match the sample size of orthodontists. Also, a helpful addition may be adding lay people to the sample size to see if they differentiate from dentists in identifying such cases to visualize the opinion of the general public.

A key limitation in our study is not including the option "cannot be determined," as many participants pointed out that they are guessing because they cannot tell if the patient had extractions made with their orthodontic treatment. This factored into our results since the participants were guessing with two options available (extraction or non-extraction), and so the theoretical probability of the correct answer by guessing is 0.5 (or 50 percent). Therefore, this third option could have been used to eliminate guessing and factor out those that in fact answered correctly, knowing from the picture whether an extraction was performed for the patient or not.

The second analysis done by only 10 participants two months after their initial contribution shows that most of their answers may be guessed with no clear mark or clue as to why they labeled the cases as extraction or non-extraction.

Another beneficial addition may be to add a rating of the attractiveness of each post-treatment photo to see the subjective rating by the participants. And so, even though the case was identified as an extraction case, the case may still have a high rate of attractiveness. In other words, the beauty and harmony of the facial esthetics are the prime concern of the orthodontist even when extractions must be performed. Previous studies showed that the “ruining” of the face is rare when the patient is diagnosed and treated correctly [9].

## Conclusions

The present study challenges the initial hypothesis that dentists are unable to identify tooth extraction based on photographs. The findings reveal that both orthodontists and dentists from other specialties demonstrated a statistically significant ability to identify these characteristics from photographic evidence.

Notably, orthodontists exhibited superior performance in identifying tooth extractions compared to their colleagues from other dental subspecialties, with a statistically significant difference between the two groups.

## Appendices

Title of the project: Distinguishing Profiles of Extraction Versus Non-extraction Cases Post-orthodontic Treatment: A Cross-Sectional Study.

The aim of the study is to assess if dentists are able to identify the gender of the patient by evaluating the dental morphology using intraoral photographs post-orthodontic treatment.

The procedures involved in this study include:

Answering 10 questions on this questionnaire using the photographs provided, which should only take you about 5 minutes to be completed.

There are no risks to you if you participate in this research. Your participation will increase knowledge about this important issue. All information collected will remain confidential. Neither your name nor address will be recorded in any assessment. There is no obligation or compulsion for you to participate, and you have the freedom to agree or not agree to participate. This will not have any effect on you. You may withdraw from the research at any time. This research does not include any medical experiments or taking biological samples.

Please indicate (✔️) below if you wish to participate or decline to do so:

I wish to participate

I do not wish to participate

Thank you for your cooperation…

Investigators’ names: Dr. Samaa Alsaraf and Dr. Hashem Alsaegh

Supervised by: Dr. Mohammad Qali

Date:                /                /                                                              Sample No.__________________

Participant’s dental speciality:    

General Practitioner Orthodontist Periodontist   

Prosthodontist Endodontist    Advanced General Dentist   

Oral Surgeon    Pedodontist Others:___________________

**Table 7:** Based on the profile photographs taken post-orthodontic treatment 1-20 provided, tick (✔️) the appropriate box

Case	Do you think this case was treated with extraction or not during their orthodontic treatment?	
	Extraction (E)	Non-extraction (NE)
1	E	NE
2	E	NE
3	E	NE
4	E	NE
5	E	NE
6	E	NE
7	E	NE
8	E	NE
9	E	NE
10	E	NE
11	E	NE
12	E	NE
13	E	NE
14	E	NE
15	E	NE
16	E	NE
17	E	NE
18	E	NE
19	E	NE
20	E	NE
